# Supplementary material for: Splice-Junction-Based Mapping of Alternative Isoforms in the Human Proteome
Source: Cell Rep. Author manuscript; Available in PMC 2020 Jan 15. (PMC6961840; doi:10.1016/j.celrep.2019.11.026)
Supplement: 2 [file NIHMS1546469-supplement-2.zip › DF1/PXD010154/Spleen/Spleen_10_BBS1_EGQSAPLLSAHVNMPGSEGLAAPNRPLNPE.pdf]

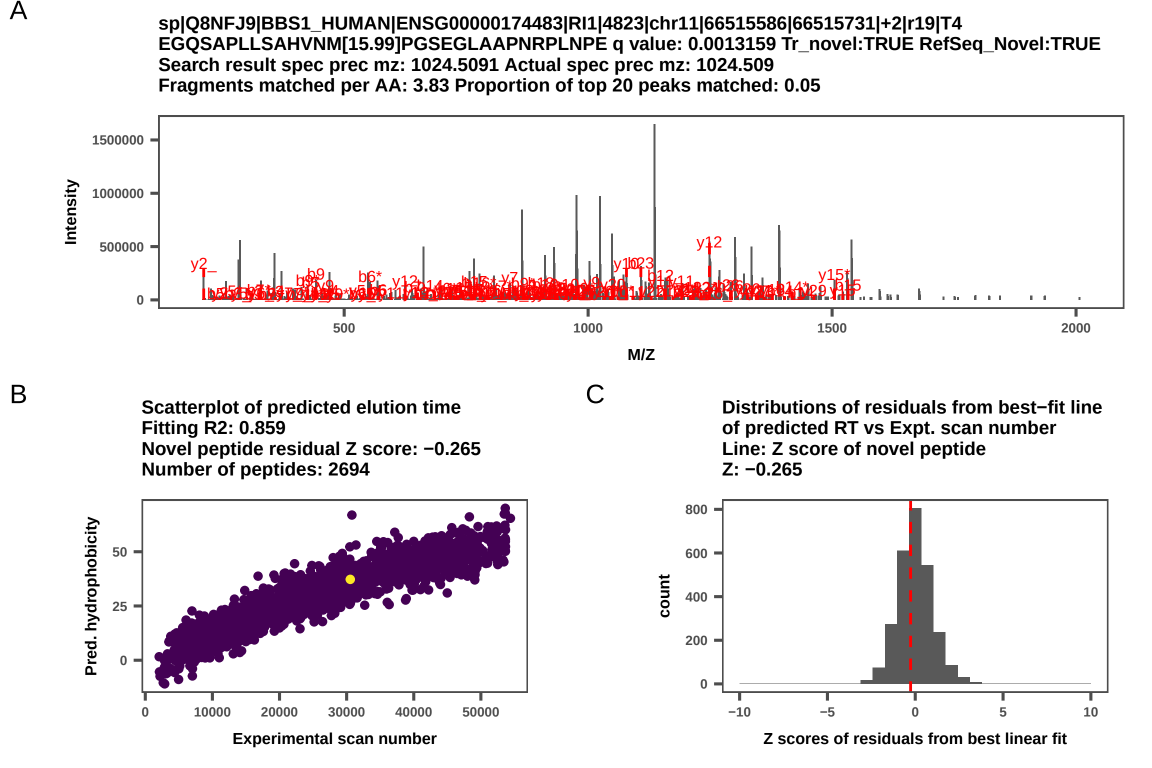

A
sp|Q8NFJ9|BBS1_HUMAN|ENSG00000174483|RI1|4823|chr11|66515586|66515731|+2|r19|T4
EGQSAPLLSAHVNM[15.99]PGSEGLAAPNRPLNPE q value: 0.0013159 Tr_novel:TRUE RefSeq_Novel:TRUE
Search result spec prec mz: 1024.5091 Actual spec prec mz: 1024.509
Fragments matched per AA: 3.83 Proportion of top 20 peaks matched: 0.05
Intensity
1500000
1000000
500000
0
500
1000
1500
2000
M/Z
y2
y12
y15*
B
Scatterplot of predicted elution time
Fitting R2: 0.859
Novel peptide residual Z score: −0.265
Number of peptides: 2694
Pred. hydrophobicity
50
25
0
0
10000
20000
30000
40000
50000
Experimental scan number
C
Distributions of residuals from best−fit line
of predicted RT vs Expt. scan number
Line: Z score of novel peptide
Z: −0.265
count
800
600
400
200
0
−10
−5
0
5
10
Z scores of residuals from best linear fit
